# Double Incomplete Internal Biliary Fistula: Coexisting Cholecystogastric and Cholecystoduodenal Fistula

**DOI:** 10.1155/2016/5108471

**Published:** 2016-01-19

**Authors:** Kemal Beksac, Arman Erkan, Volkan Kaynaroglu

**Affiliations:** Department of General Surgery, Hacettepe University Medical School, Sihhiye, 06100 Ankara, Turkey

## Abstract

Internal biliary fistula is a rare complication of a common surgical disease, cholelithiasis. It is seen in 0.74% of all biliary tract surgeries and is thought to be a result of repeated inflammatory periods of the gallbladder. In this report we present a case of incomplete cholecystogastric and cholecystoduodenal fistulae in a single patient missed by ultrasonography and endoscopic retrograde cholangiopancreatography and diagnosed intraoperatively. In the literature there is only one report of an incomplete cholecystogastric fistula. To our knowledge this is the first case of double incomplete internal biliary fistulae.

## 1. Introduction

Cholelithiasis is a common surgical problem. It is usually treated laparoscopically and resolves without complication. Cholecystoenteric fistula, which may occasionally be multiple, is a rare long term complication of gallstone disease [1]. It has been reported in 0.74% of patients undergoing biliary tract surgery [2, 3]. Cholecystoduodenal fistula is the most common form (53%) while cholecystogastric fistula is the rarest (3%) [4]. Incomplete internal biliary fistula has been reported only once in 1955 by Chamberlain [5]. In this report, we present a case of double incomplete internal biliary fistula, cholecystogastric, and cholecystoduodenal, which to our knowledge is one of its kind.

## 2. Case Report

A 69-year-old male was admitted to hospital for one-week history of abdominal pain and jaundice. He denied previous periods of abdominal pain, acholic gaita, and weight loss. His past medical history included diabetes mellitus, regulated by oral antidiabetics and myocardial infarction followed by 3 cardiac stent placements.

His physical examination was remarkable for jaundice and tenderness in right upper quadrant. He did not show any evidence of guarding or rebound tenderness and was negative for Murphy's sign. His vital signs were within normal range.

Laboratory workup revealed slight anemia (Hb: 12.8 g/dL) and elevated liver enzymes (ALT: 601 U/L, AST: 205 U/L, GGT: 623 U/L, and ALP: 279 U/L) and high bilirubin level with direct bilirubin dominance (total bilirubin: 1.35 mg/dL, direct bilirubin 0.93 mg/dL). Abdominal sonography showed multiple millimetric gallstones in the gallbladder. Endoscopic retrograde cholangiopancreatography (ERCP) was performed due to his jaundice. During ERCP, common hepatic duct and the choledoch duct were found to be dilated and two millimetric gallstones were extracted from distal choledoch following papillotomy.

**Figure 1:**
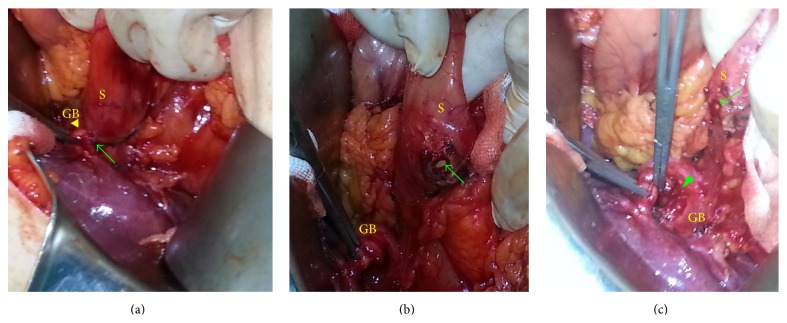
Incomplete cholecystogastric fistula. (a) Fistula tract between the gallbladder and stomach. (b, c) Appearance after fistulectomy. The arrow indicates the fistula opening on the stomach wall. The arrowhead indicates the fistula opening on the gallbladder (GB: gallbladder and S: stomach).

**Figure 2:**
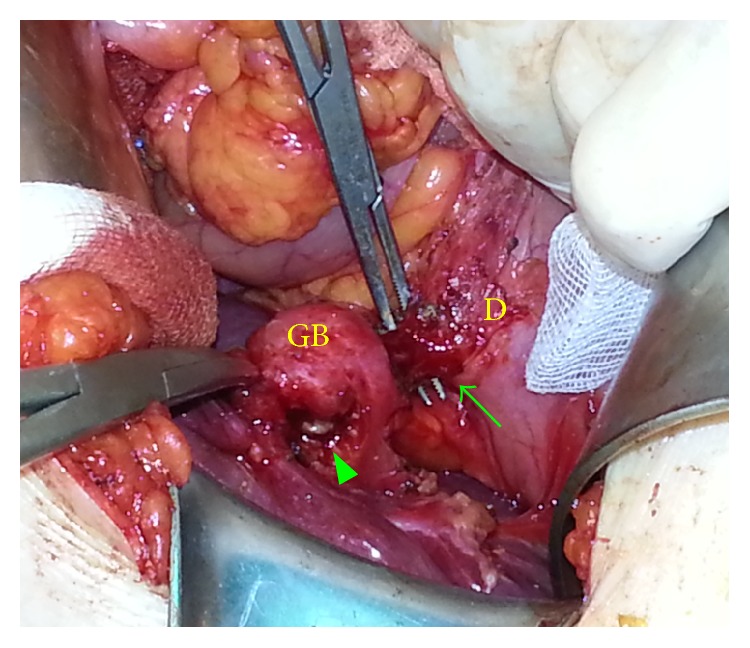
Incomplete cholecystoduodenal fistula. The arrow indicates the cholecystoduodenal fistula. The arrowhead indicates the cholecystogastric fistula opening on the gallbladder, which was previously excised.

Six weeks after ERCP, he was taken to the operating room with normal liver function tests. Laparoscopic exploration revealed dense adhesions between the gallbladder, stomach, and duodenum. It was converted to open surgery due to difficulty in dissection. After meticulous adhesiolysis, two fistula tracts, cholecystogastric and cholecystoduodenal, have been identified. First the cholecystogastric fistula was excised and a 1 cm gallstone was extracted from the stomach wall (Figure 1). When a clamp was advanced through the opening to explore for more stones it has been discovered that the fistula had not reached the mucosa yet and the fistula was incomplete. To verify this, nasogastric tube was advanced beyond the fistula site and it passed into duodenum easily. The same procedure was repeated for the cholecystoduodenal fistula and it was also found to be an incomplete fistula (Figure 2). The seromuscular defects were repaired primarily and cholecystectomy was performed. Postoperative course was uneventful. There was no evidence of biliary leak. Patients' vital signs and blood count analysis were normal. He started oral feeding on postoperative day two. His drains were removed the next day and the patient was discharged successfully.

## 3. Discussion

Internal biliary fistula is an uncommon surgical pathology primarily affecting geriatric population [4]. It is usually diagnosed intraoperatively unless a unique symptom such as gastric outlet obstruction (as in Bouveret's syndrome) or vomiting gallstones is present. ERCP is the gold standard modality for the diagnosis of biliary fistula [6–8]. The current patient also had preoperative ERCP; however, the fistulae were missed due to an uncommon presentation. Since the fistulae were incomplete, the gallstones were stuck in the muscular layer of the stomach and duodenum wall. That is why the fistulae could not be seen by the endoscope. That is also why the contrast substance could not pass into the lumen and show the fistula tract. A bilioenteric fistula may cause gallstone ileus, Bouveret's syndrome, and gastrointestinal bleeding due to the presence of the stone in the alimentary tract. Pneumobilia may be significant in patients without a history of bilioenteric anastomosis [9, 10]. None of these symptoms were present in our patients as the fistula tract was incomplete. A history of jaundice has been reported in 50% of patients, which was also the case in the patient [11].

Majority of spontaneous internal biliary fistulae (91–94%) result from gallstone disease [12]. Less common causes are peptic ulcer disease and neoplasms of the stomach, gallbladder, pancreas, duodenum, jejunum, colon, or bile ducts [6, 13]. According to Glenn, after stone formation, an inflammatory process results in adhesions between gallbladder and adjacent viscus. Repeated periods of such inflammatory process result in the destruction of the wall of gallbladder and adjacent viscus ultimately resulting in fistula formation [10, 14]. Our patient does not only support this theory but also shows us the ongoing process of fistula formation. He had enough amount of inflammation for the gallstones to penetrate the gallbladder but not enough to penetrate through the mucosa yet.

In conclusion, cholelithiasis and laparoscopic cholecystectomy are one of the most common clinical conditions and performed operations in daily general surgery practice. Although both are well known to surgeons, there may be still surprising complications. Internal biliary fistula should be kept in mind while taking down the adhesions around the gallbladder.
